# Recent Progress on Protein-Polyphenol Complexes: Effect on Stability and Nutrients Delivery of Oil-in-Water Emulsion System

**DOI:** 10.3389/fnut.2021.765589

**Published:** 2021-11-02

**Authors:** Minghui Li, Christos Ritzoulis, Qiwei Du, Yefeng Liu, Yuting Ding, Weilin Liu, Jianhua Liu

**Affiliations:** ^1^College of Food Science and Technology, Zhejiang University of Technology, Hangzhou, China; ^2^Department of Food Science and Technology, International Hellenic University, Thermi, Greece; ^3^School of Food Science and Biotechnology, Zhejiang Gongshang University, Hangzhou, China; ^4^Hangzhou Huadong Medicine Group Pharmaceutical Research Institute Co. Ltd., Hangzhou, China

**Keywords:** protein, polyphenol, interaction, oil-in-water emulsion, antioxidant, stability, nutrient delivery, ligand

## Abstract

Oil-in-water emulsions are widely encountered in the food and health product industries. However, the unsaturated fatty acids in emulsions are easily affected by light, oxygen, and heat, which leads to oxidation, bringing forward difficulties in controlling emulsion quality during transportation, storage, and retail. Proteins are commonly used as emulsifiers that can enhance the shelf, thermal and oxidation stability of emulsions. Polyphenols are commonly found in plants and members of the family have been reported to possess antioxidant, anticancer, and antimicrobial activities. Numerous studies have shown that binding of polyphenols to proteins can change the structure and function of the latter. In this paper, the formation of protein–polyphenol complexes (PPCs) is reviewed in relation to the latters' use as emulsifiers, using the (covalent or non-covalent) interactions between the two as a starting point. In addition, the effects polyphenol binding on the structure and function of proteins are discussed. The effects of proteins from different sources interacting with polyphenols on the emulsification, antioxidation, nutrient delivery and digestibility of oil-in-water emulsion are also summarized. In conclusion, the interaction between proteins and polyphenols in emulsions is complicated and still understudied, thereby requiring further investigation. The present review results in a critical appraisal of the relevant state-of-the-art with a focus on complexes' application potential in the food industry, including digestion and bioavailability studies.

## Introduction

Oil-in-water emulsions are widely used in the food industry and often play specific functions such as flavor carriers or as functional substances. Functional oils in oil-in-water emulsions (fish oil, algal oil, etc.) are rich in polyunsaturated fatty acids such as eicosapentaenoic acid (EPA) and docosahexaenoic acid (DHA), which are beneficial for the health of elderly and young children. These oils have health functions such as lowering cholesterol and promoting brain development. However, oil-in-water emulsion systems are generally unstable, as they are prone to oxidation, stratification, breakage and other phenomena in the process of storage, transportation and processing, making the emulsions' quality decline. The instability of unsaturated oils in oil-in-water emulsion systems is mainly caused by oxidation, and the oxidation itself is mainly due to three processes: autoxidation, photosensitive oxidation and enzymatic oxidation; of these the autoxidized chain reaction is the most significant (1). Free radicals are mainly responsible for the oil automatic oxidation. Free radicals are mainly derived from normal metabolic processes in cells and generated by the reaction between metal ions (cobalt, copper, iron, etc.) and oil during the processing (2). The outer layer of the free radical electron shell has an unpaired electron, which has a strong affinity to free electrons, so it can play the role of a strong oxidant. The chain reaction of free radicals causes the auto-oxidation of oils and fats. When the oil in the emulsion is autoxidized, the stability of the emulsion decreases and the flavor deteriorates, resulting in stratifying and other phenomena (3). Therefore, it is necessary to improve the oxidative stability of oil-in-waters emulsion in the application process of food industry.

Efficient emulsifiers can form a layer on the oil-water interface of droplets and protect again coalescence, hence increase the storage stability of the emulsion. Proteins are often used as emulsifiers to improve emulsion stability. Some milk protein-derived proteins, such as whey proteins, can form interfacial layers able to offer protection against not only coalescence, but also to inhibit the oxidation of oil-in-water emulsions during storage and transportation (4).

Some milk proteins have strong antioxidant ability. In oil-in-water emulsions, the antioxidant capacity of interfacial emulsifiers could have a conspicuous influence on the rate of lipid oxidation by affecting the reactivity and location of the pro-oxidative transition metals, lipid hydroperoxide and free radicals (5, 6). For example, caseins have been shown to have significant antioxidant properties in oil-in-water emulsions, and the antioxidant activity of casein has mainly been attributed to its ability to bind pro-oxidants (7).

Polyphenols have antibacterial, anti-cancer, cardiovascular disease-preventive, anti-oxidative, and other health-related and functional properties (8). However, phenolic compounds are apt to oxidize to quinones under oxygen, ozone, or polyphenol oxidase (9). Therefore, polyphenols have strong antioxidant properties but poor emulsifying ability. The interaction between polyphenols and proteins improves protein antioxidant activity and also broadens the application of polyphenols. A previous research reported that, compared to egg white protein, egg white protein (EWP)-epigallocatechin gallate (EGCG) complexes could significantly improve the emulsifying properties and the stability of emulsions (10). Proteins, interacting covalently and/or non-covalently with polyphenols, could change the structure and function of the former, which affects their role as emulsifiers in oil-in-water emulsions. Milk proteins α-casein (α-CS) and β-CS interactions with tea polyphenols were analyzed by spectral analysis (identifying substances and determining their chemical composition and relative content) and docking experiments (studying the interaction between molecules). It was found that the interactions change the secondary structure of proteins and improve their antioxidant activity, which is consistent with the previous cases (11).

The present paper reviews the formation of complexes between proteins and polyphenols through covalent and non-covalent interactions. Both these modes of action induce PPCs (protein-polyphenol complexes) to have good interfacial properties on the surface of the emulsion. For example, PPCs can form a thick film on the surface of the oil droplets, which can prevent the oxidation of the oil droplet and improve the stability of the emulsion. Therefore, they can be used as emulsifying agents that enhance the oxidation stability, emulsification stability and thermal stability of oil-in-water emulsions. Moreover, after PPCs are formed, the particle size and electric potential are reduced, making it potentially easier to attach to the surface of oil droplets and be used to resist enzymes in the stomach environment, and then allow them to slowly release in the intestines. Of course, their relevant effectiveness in the presence of bile salts remains to be examined. In addition, the emulsions can be used to carry bioactive substances in oil, so the oil droplets loaded with PPCs will also be more conducive to the transportation and release of active substances.

To sum up, understanding the interactions between protein and polyphenol offers the potential to substantially improve the mode of utilization of proteins in the food industry, and their applications in stabilizing oil-in-water emulsions can open new possibilities in functional food design. PPCs also have a great significance in improving the storage stability of the oil-in-water emulsions, and their application in the food industry.

## Protein–Polyphenol Complexes (PPCs)

### Formation Mechanisms of Protein–Polyphenol Complexes

The interactions between protein and polyphenol can be divided into reversible interactions and irreversible interactions. Reversible interactions are carried out by non-covalent bonds such as hydrogen bonds, hydrophobic bonds and van der Waals forces, while irreversible interactions are generally the ones where proteins and polyphenols combine into complexes by covalent bonding, thus affecting the respective structural properties of proteins and polyphenols (12). Among the two kinds of interactions, the non-covalent interactions are the most abundant in nature. Interactions involving non-covalent bonds are susceptible to environmental factors, such as changes in temperature and pH, which will affect the binding of protein and polyphenols.

### Covalent Interactions

**Figure 1:**
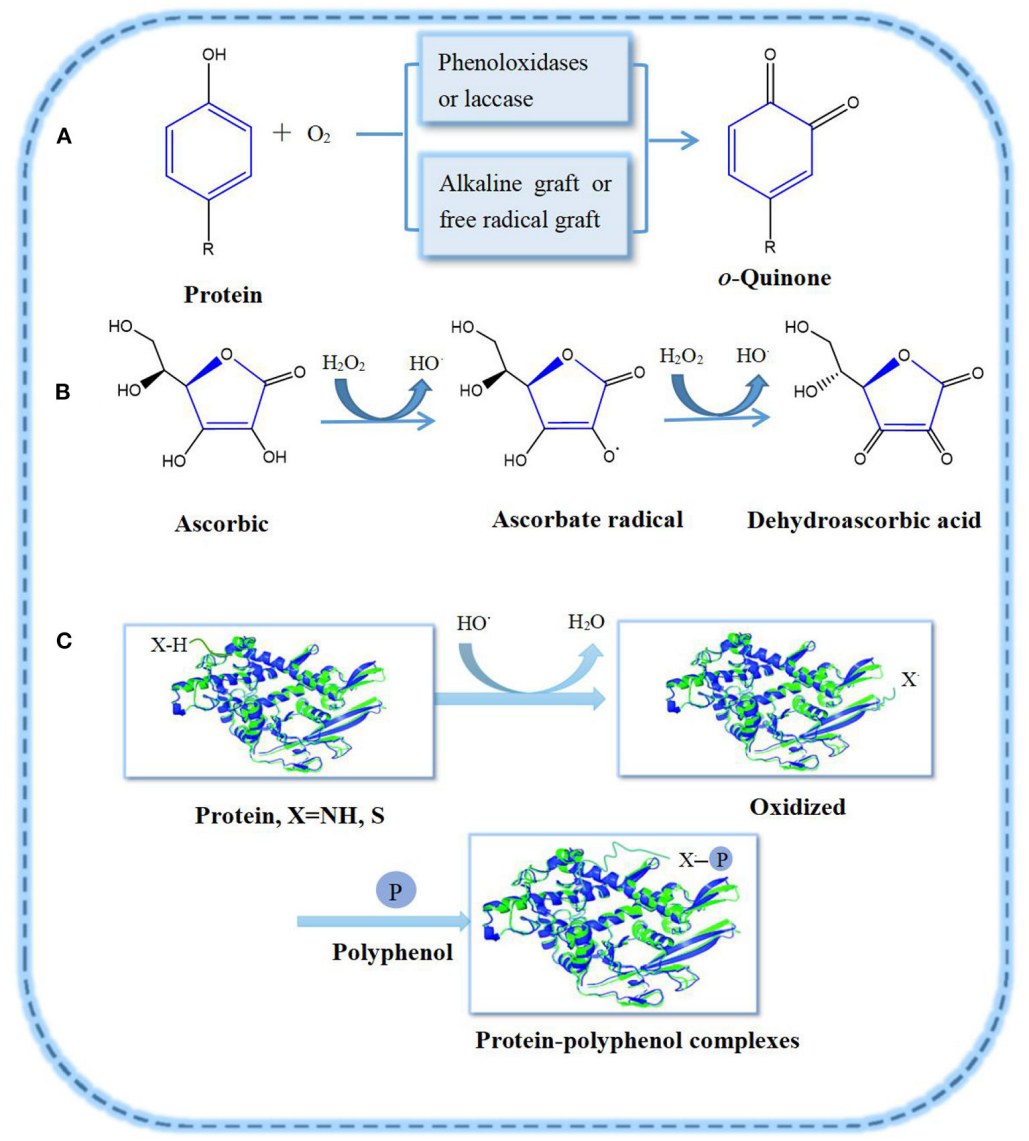
Oxidation of polyphenols and their covalent graft reactions with proteins: **(A)** Polyphenol oxidation pathway; **(B)** Ascorbic acid oxidation system; **(C)** Protein-polyphenol radical grafting reaction.

The covalent interactions between proteins and polyphenols are usually through C–N or C**–**S linkage (Figure 1). Under alkaline conditions, polyphenols form the corresponding quinones in the presence of enzymes and oxygen (13). In the process of forming PPCs, the polyphenols' enzymatic oxidation intermediate products, such as half quinone radicals of nucleophilic residues, interact with amino acids (e.g., methionine, lysine) on the protein side chain. Finally, protein and polyphenol form covalent bonds (14). If polyphenols are partially oxidized and then combined with proteins, they may also form complexes with proteins, at times improving the latter's emulsification and antioxidant properties (15). For example, oxidized tannin (TA) and catechin acid (CA) can covalently combine with porcine plasma proteolytic products as to form complexes under alkaline conditions, improving the emulsifying and antioxidant properties of polypeptides (9).

Proteins are reported to bind to polyphenols by enzymatic and non-enzymatic methods (3, 15). Non-enzymatic methods mainly involve free radical grafting, hydrogen peroxide and ascorbic acid as redox systems. The free radical grafting process involves proteins binding to polyphenols and is considered as a better method to produce high active PPCs, as it can improve the antioxidant activity of proteins more efficiently than the alkaline method (3). Feng et al. (16) grafted ovalbumin (OVA) with catechins such as (+)-catechin (C), (-)-epigallocatechin (EGC) and (-)-epigallocatechin gallate (EGCG) as to form covalent complexes. The binding of OVA to polyphenols was confirmed by sodium dodecyl sulfate-polyacrylamide gel electrophoresis (SDS–PAGE) and matrix-assisted laser desorption/ionization time-of-flight mass spectrometry (MALDI–TOF–MS). Covalent interaction of gelatin-gallic acid/catechin (gelatin–GA/CT), β-lactoglobulin–catechin (β-LG–CT), α-lactalbumin–CT, and lactoferrin–chlorogenic acid/epigallocatechin gallate/gallic acid (LF–CA/EGCG/GA) were successfully prepared *via* this method, and the effect of polyphenol modification on the structural and functional properties of these proteins have been estimated (17–19).

### Non-covalent Interactions

**Figure 2:**
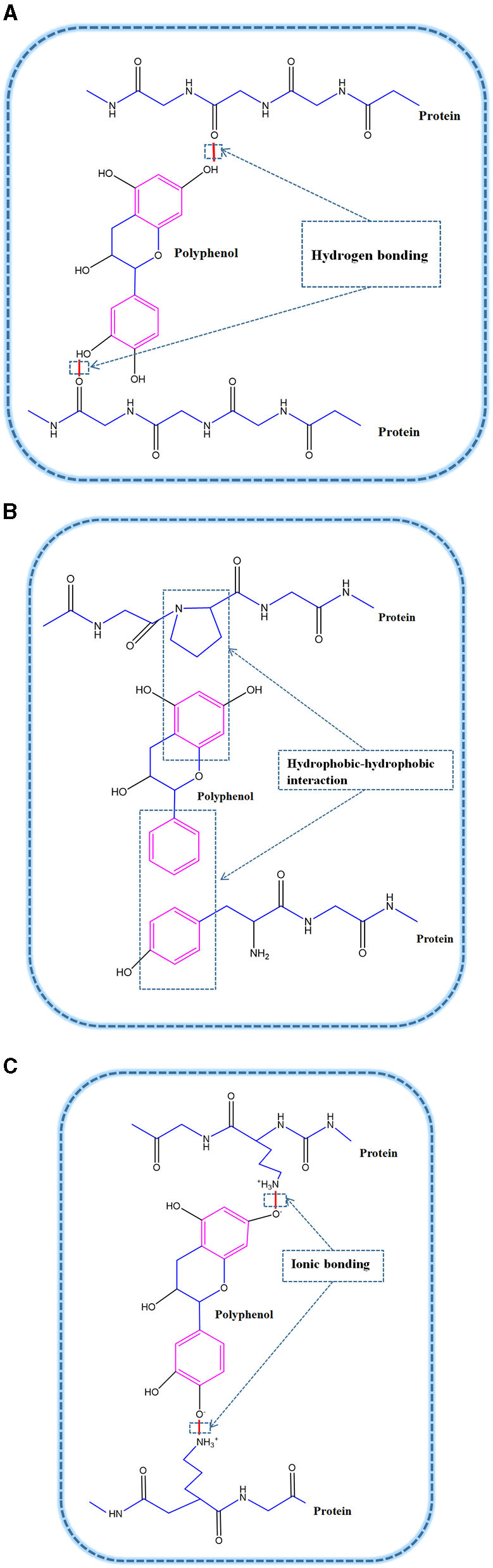
Non-covalent interactions between proteins and polyphenols: **(A)** Hydrogen bonding; **(B)** Hydrophobic-hydrophobic interaction; **(C)** Ionic bonding.

The non-covalent interactions (Figure 2) between protein and polyphenols mainly occur via hydrogen bonds, hydrophobic interactions and electrostatic interactions (20). Hydrogen bonding and hydrophobic interactions are the main forces involved in the non-covalent synthesis of complexes between proteins and polyphenols (20, 21).

#### Hydrogen Bonds

The polyphenols can be used as hydrogen donors to form a hydrogen bond with the C = O group of the protein (22). When interacting with proteins, as long as hydrogen bonds are formed through carbon-oxygen double bonds, interaction forces between proteins and polyphenols are exerted, resulting in bonding. Besides, hydrogen bonds also form via the interactions between the OH groups of polyphenols and oxygen or nitrogen, especially the hydroxyl (–OH) and amino (–NH_2_) groups of proteins (23).

It has been shown that casein residues (Phe23, Phe24, Phe28, Phe32, and Val31) can interact with catechins via hydrogen bonds (24). In addition, the main mode of interaction between plant proteins such as pea protein and grape seed pro-anthocyanidins is hydrogen bonding, and the interaction between pea protein and pro-anthocyanidins can make emulsion more stable (25), which also extends the application field of non-covalent interactions between protein and polyphenols in emulsion systems.

#### Hydrophobic Interactions

Surface hydrophobicity (*H*_0_) is an important parameter affecting the surface-related functions of proteins (26). The hydrophobic interaction between proteins and polyphenols is one of the main forces involved in the formation of PPCs, hence plays an important role in the formation of non-covalent protein–polyphenol complexes. Several researchers, such as Yuksel et al. (27), Hasni et al. (11), and Staszawski et al. (28), reported that the interaction between proteins and polyphenols is mainly accomplished through hydrophobic forces. They also reported that cytochrome adsorbed on a gel column containing immobilized polyphenols could be eluted by anion and non-anion eluents, thus proving that hydrophobic interaction between proteins and tannin acid (TA) may be influenced by surfactants.

In addition, studies have shown that some hydrophobic amino acids, such as leucine and glycine, can interact with the non-polar aromatic rings of polyphenols, highlighting the nature of the main PPCs hydrophobic interactions (12, 29). Through docking studies, Kanakis et al. (29) found that tea polyphenols had a weak binding hydrophobic interaction with β-lactoglobulin (LG) in emulsions. In addition, they discovered that the hydrophobic interaction of polyphenols with β-LG altered the secondary structure of the protein (29), making it more stable, a property beneficial to the stability of relevant PPCs-stabilized emulsions. Therefore, hydrophobic interactions between proteins and polyphenols can change the structure and interfacial environment of proteins.

#### Ionic Binding Forces

Amino acids are the structural units of proteins, which possess different electrically charged, uncharged polar, and hydrophobic side chains. The charges side chains may interact with polyphenols via ionic binding interactions (30). The ionic bond and force interaction between proteins and polyphenols are mostly due to the attractions between the positively-charged amino acids, such as lysine, and the negative-hydroxyl groups of polyphenols. For example, Han et al. (30) reported that lysine, having a higher positive charge than hemoglobin or immune protein, demonstrated stronger ionic interactions with catechin (CT). Therefore, the degree of ionic interaction between proteins with different side chain groups and polyphenols is different, which will further influence the functional differences of protein-polyphenols complexes. Of course, as the charges of proteins and (conditionally) of PPCs are strongly dependent on pH, these interactions are pH-specific.

## Effects of Protein–Polyphenols Interaction on the Protein Structure and its Functional Properties

### Changes in Protein Structural Properties

When proteins interact with polyphenols to form PPCs, the secondary, tertiary and quaternary structures of proteins can be affected. The polyphenols can change the secondary conformation of proteins mainly by the conversion between α-helical, β-sheet, β-turn, and random coil (11). Ge et al. (31) used circular dichroism (CD) to analyze the secondary structure of soybean soluble protein bound with tea polyphenols. They found that tea polyphenols increased the content of α-helix and the loose structure of random coils disappeared, which contributed to a stabler conformation of the protein (31). Liu et al. (32) analyzed and determined the structure of lactoferrin (LF) after coupling with polyphenols and measuring by CD. Moreover, they also found that the wavelength band of far-UV caused by conjugation and the negative peak-to-peak position related to α-helix increased. The complexes of LF and CA or EGCG caused a small change in the band intensity from 210 to 230 nm. It can be concluded that the conjugation of LF with polyphenols gave rise to an increased fraction in α-helix with a parallelly-decreasing fraction in the random coil structure, indicating restructure behavior effect on LF. Nevertheless, it was reported that the interaction of CA with bovine serum albumin (BSA) would cause a decreased fraction of α-helix together with increased portions of other structures, which might be influenced by the differences in the physicochemical characteristics between LF and BSA, as well as by the methods used to prepare these conjugates (13).

Information can be obtained about the conformational and/or dynamic changes of proteins during their interaction with phenolics by fluorescence spectroscopy analysis. The structures of LF–EGCG, LF–CA, and LF–GA complexes were measured by fluorescence spectrophotometer, and it can be concluded that the conjugation phenomenon of LF-polyphenol complexes may change the tertiary structure of LF (32). Usually, the maximum emission wavelength of LF is 342 nm, while the measured maximum emission wavelength of LF–EGCG, LF–CA, and LF–GA are 351, 345, and 344 nm, respectively (32). The fluorescence intensity of the interaction between LF and polyphenols decreased significantly, and the maximum emission wavelength showed a red shift caused by the transfer of tryptophan residues to a more hydrophilic environment. It could be deduced that the interaction between LF and polyphenols induced conformational changes that might lead to the unfolding and denaturation of LF. It should be stressed that the derivatization of polyphenols will also affect the protein. Klaus et al. (33) showed that the binding of derivatization chlorogenic acid (CA) to β-LG affected the protein amino acid composition in experiment involving rats; while high derivatization level could affect the nutritional quality of β-LG. It might be the concentration of the reactants that causes the structural change. An explanation for this is that the interaction between protein and polyphenols is affected by the type of protein, or by differences between *in vitro* and *in vivo* experiments (33). Therefore, the effect of polyphenols on proteins is different on a case-by-case basis: Some proteins are not affected by the interaction with polyphenols, while they are influenced by external factors like the concentration of the reaction solution.

### Changes in Protein Functional Properties

Protein–polyphenol complexes not only affect the structure of proteins but also change their functional properties. For example, the interactions between proteins and polyphenols can improve their emulsification properties and oxidation resistance capacity.

The interactions between proteins and polyphenols can significantly increase the stability of protein-based colloid systems and protein-stabilized emulsions (34). Liu et al. (35) showed that the average diameter and polydispersity index of the droplets coated with LF–polyphenol complexes were less than those coated with LF alone. According to this report, the emulsifying properties of LF have been greatly improved after being covalently modified by EGCG. Staszewski et al. (6, 28) also showed that the stability of oil-in-water emulsion emulsified by sunflower protein–CA complexes was higher than that of plain sunflower protein. These might be because the combination of polyphenols with LF increases its surface hydrophobicity and enhances its surface activity, and/or leads to the formation of a thicker interfacial layer. Other research also showed that the improvement of emulsification performance might be due to the increase of protein flexibility, solubility, and surface hydrophobicity, thus improving the ability of protein adsorption on oil-water surfaces (36).

Whey protein is one of the main proteins of milk, has good emulsifying properties and is widely used in the food processing industry (37). Whey protein can stabilize emulsions on its own, while studies have shown that it has a strong non-covalent affinity to tannic acid (TA), leading to complexes that will not only affect the secondary structure of the protein, but will also reduce the stability of emulsions (38). However, other studies have shown that the covalent and non-covalent interactions between TA and other proteins, such as gliadin, can improve the emulsification ability of the protein (39), thus enhancing the stability of the emulsion produced with the relevant PPCs. These are cases where the non-covalent interactions between protein and polyphenol are not good for the stability of oil-in-water emulsions, while covalent binding between protein and polyphenol is beneficial to the stability of emulsions, and has a profound influence on the latter's during storage. Of course, such examples relate to very few proteins and should not be generalized. It should be noted that phenolic acids and flavonoids reacting with soy protein may destroy amino acids (Lys, tryptophan) and amino acid residues (cysteine residues) in protein molecules, thus affecting the availability of the protein's amino acids (13).

Antioxidant activity is one of the most important properties of protein–polyphenol complexes. The following studies concluded that protein–polyphenol complexes exhibited stronger antioxidant activity than the unmodified proteins. Sun et al. (40) showed that the antioxidant capacity of EWP was significantly improved when coupled with catechin (CT) and CA. Studies have shown that under the same protein concentration, the DPPH free radical scavenging capabilities of EWP-CT and EWP-CA are 38.0 and 67.7%, respectively, which were 2.5- and 4.5 fold than that of unmodified EWP (40). As reported earlier, Staszewski et al. (6) also concluded that the combination of tea polyphenols with β-LG could improve the oxidation stability of oil-in-water emulsions, while the complexes had stronger antioxidant activities than the pure β-LG. This can be attributed to the fact that the compound-stabilized emulsion had lower concentrations of hydroperoxide, which can account for its better stability (6). You et al. (41) also found that 2,2'-azino-bis (3-ethylbenzothiazoline-6-sulfonic acid) (ABTS) radical scavenging activity, ferric reducing activity power (FRAP), and oxygen radical absorbance capacity (ORAC) of ovotransferrin–CT complexes were increased by 4–5 times as compared to those of native ovotransferrin (14). In addition, Feng et al. (16) revealed that complexes of ovalbumin (OVA) with EGCG, EGC, or CT synthesized by free-radical grafting showed stronger ORAC, 1,1-diphenyl-2-picrylhydrazyl (DPPH) and ABTS radical scavenging activities than OVA alone. Gu et al. (42) measured the antioxidant activities of EWP, of the mixture of EWP and CT, and of the EWP–CT complexes by reference to the previous ABTS free radical scavenging ability experiment, and the results showed that the antioxidant activity of EWP–CT complexes were much higher than those of pure EWP. Moreover, the complexes of EWP and tea polyphenols could enhance the antioxidant activity of EWP (43). In this work, the DPPH radical scavenging activity of EWP was significantly improved (*P* < 0.05) after covalent conjugating with TP alkaline/free radical methods (44). In addition, the thiobarbituric acid reactive substance (TBARS) of FPI and FPI–FPP were measured in the design of the antioxidant performance test of PPCs, and the results of 15 days-old specimens showed that the TBARS of the FPI–FPP complexes were significantly lower than that of FPI (34). Furthermore, the antioxidant capacities of PPCs were improved because of the introduction of hydroxyl groups from polyphenols into proteins (23, 41, 43). Therefore, protein–polyphenol complexes can be used to enhance antioxidant properties with an aim to improve the oxidative stability of several lipid-based foods.

Many foods need heat treatment such as sterilization, pasteurization, and cooking in the process of manufacture or use, so it is useful to detect the effect of heating on the stability of emulsions (35). The addition of polyphenols affects the thermal stability of the protein. For example, after free radical grafting of LF with EGCG, CA, and GA, respectively, the melting temperature of LF increased from 5 to 15.2°C (32). The melting temperature of milk protein also increased with the addition of EGCG, which improved the stability of the produced emulsion (45). They also verified that covalent complexes had better thermal stabilities than non-covalent complexes, as shown by the thermal decomposition temperature (T_d_). This suggested that covalently modified milk proteins needed less energy to unfold, because they are more stable. This might be due to the spatial expansion of milk protein in the chemically modified part of EGCG. In addition, studies showed that the stability of emulsion stabilized with LF was low when compared to that of LF–CA complex emulsions (35). This might be due to the spatial expansion of LF above the thermal denaturation temperature, resulting in a strong hydrophobic force between lipid droplets. As a result, the droplets flocculated/coalesced. However, the emulsion coated with LF–CA was highly stable to high temperature processing. Differential scanning calorimetry (DSC) measurements of protein thermal behavior showed that the denaturation temperatures of LF–CA complexes were higher than that of natural LF.

The interaction between proteins and certain polyphenols will also affect the functional properties of proteins due to structural differences between the different polyphenols and the relevant interactions. For example, some researchers have reported that egg protein and tea polyphenol complexes can increase the resilience of air/water surfaces and enhance foamability (46). In addition, Li et al. (36) showed that the binding between LF and procyanidins improved the foaming properties of LF, which could be used to improve the quality-related properties of natural functional foods. Other researchers considered tannic acid (TA) and caseinate (CS) complexes, and found that complexes would actually decrease their foamability (47).

## Types of Protein–Polyphenol Complexes

### Animal Proteins and Polyphenols

Animal protein mainly comes from poultry, livestock and fish meat, and from eggs and milk. The dominant proteins are the caseins (extracted from milk), which can be better absorbed and utilized by adults (48). Compared with plant protein, animal protein has more essential amino acids. The interactions between animal proteins (such as the previously-mentioned EWP, CN, OVA and LF) and polyphenols (such as CA, CT, and EGCG) not only enhanced most of the studied functional properties of protein (Table 1), but also expanded the application of animal proteins in food and cosmetic industry. For example, EWP has a high surface activity and is often used as a foaming agent in food industry (50). Quan et al. (14) also showed that EWP enhances emulsion stability, and is often used in food as an emulsifier. In addition, Sun et al. (40) found that the covalent complexes between EWP and polyphenols (CT or CA) can significantly improve the antioxidant activity and emulsifying performance of EWP, thus improving the oxidative and coalescence stability of emulsions. Moreover, LF is often used to interact with polyphenols, polysaccharides or other proteins to improve its emulsifying properties. Recent reports suggested that the complexes formed by LF and some reactive small molecules such as polyphenols can improve the emulsion antioxidation properties (51). Furthermore, Liu et al. (32) calculated the emulsifying activity index (EAI) and emulsion stability index (ESI) parameters of the unmodified LF and LF–polyphenol complexes, and the results showed that the ESI of LF–EGCG was improved and the emulsion stability was higher than that of LF (32). In addition, gelatin–anthocyanin complexes can change the functional properties of hydrophilic gelatin proteins and thus improves the stability of emulsions (49).

**Table 1:** Typical animal protein-polyphenol complexes.

**Complexes**	**Combining with the way**	**Characterization methods**	**Functions**	**References**
EWP/CA/CT	Covalent: free radical graft; alkaline methods	Circular dichroic (CD) spectroscopy; fluorescence analysis	Improving antioxidant activity and emulsifying performance	(40, 44)
LF-EGCG	Covalent: free radical graft	FTIR spectroscopy; DSC measurement; fluorescence spectroscopy	Improving solubility and emulsifying properties	(32)
Gelatin-Anthocyanin	Non-covalent interaction: hydrogen	Circular dichroism (CD); isothermal titration calorimetry (ITC)	Improving antioxidant activity	(49)

### Plant Proteins and Polyphenols

The use of plant protein to replace animal protein is becoming the trend of more and more food companies (Table 2). Because of its good surface activity, pea protein can be used as a natural emulsifier for application in emulsions; however, it was shown to exhibit inferior oxidation inhibition effect compared to other proteins (48). Other studies reported that the interactions between pea protein and proanthocyanidins can improve the antioxidant ability of pea protein, thus improving the stability of oil-in-water emulsions (25). Rice bran protein (RBP), composed of albumin, globulin, gliadin, and glutenin, is a complete protein. However, most of rice bran is still used as animal feed, which is a serious waste of resources. RBP–CT complexes can affect the structural and functional properties of RBP, improve its emulsifying performance, and thus enhance the stability of relevant oil-in-water emulsions (36). The study showed that the addition of CT reduced the incidence of RBP α-helix and β-sheet. The surface hydrophobicity of RBP was enhanced and the interfacial tension between oil and water was decreased. In addition, the emulsion stabilized by RBP–CT had smaller particle size and better emulsifying properties (36).

**Table 2:** Typical plant and microbial protein-polyphenol complexes.

**Complexes**	**Combining with the way**	**Characterization methods**	**Functions**	**References**
OVA-CT/EGC/EGCG	Covalent: free radical graft	Circular dichroic(CD) spectroscopy; DSC measurement; FTIR spectroscopy	Improving antioxidant activity and interfacial accumulation	(16)
PP-PC	Non-covalent interaction: hydrogen; hydrophobic interactions	Isothermal titration calorimetry analysis; molecular docking	Antioxidants	(25)
RBP-CC	Non-covalent interaction: hydrogen; hydrophobic binding	FTIR spectroscopy	Higher viscosity and viscoelasticity; Increased hydrophobicity	(36)
Lysozyme-Proanthocyanidins	Non-covalent interaction: hydrogen	Gel permeation chromatography	Improve protein foam stability	(21)

### Microbial Proteins and Polyphenols

Generally, microorganisms can use sugar, volatile fatty acids, and carbon dioxide and nitrogen sources to form amino acids. Under the appropriate energy supply, the amino acids are then converted to microbial proteins (Table 2). Microorganisms contain a multitude of enzymes that can break down food, so microbial proteins are often present in the form of enzymes. Microbial proteins have a high affinity to polyphenols (such as proanthocyanidins) at a suitable pH. Lysozyme is a spherical food protein (52). Prigent et al. (21) showed that the affinity between lysozyme and proanthocyanidins was strong at higher pH, and the stabler form of lysozyme–proanthocyanidins complexes appeared at higher pH (pH 7.5). In addition, microbial proteins can be produced thousands of times more efficiently than in animals or in plants (53), hence the microbial protein–polyphenol materials may have wider development prospects in the food industry.

## Effect of Protein–Polyphenol Complexes on Stability of Emulsions

### Zeta-Potential and Particle Size

The protein adsorbed on the surface of the oil droplets in an oil-in-water emulsion may provide charges on the surface of oil droplets. The ζ-potential is a measure of the surface charge density of a particle, which typically relates to the ζ-potential of the emulsion system onto whose droplets the proteins are adsorbed (54). Li et al. (36) measured the ζ-potential of RBP and RBP–CT complexes and found that it first increased and then decreased with increasing polyphenol concentration. When the polyphenol concentration was 0.15 (%, W/V) and protein concentration was 0.1 (%, W/V), the ζ-potential reached its maximum value. RBP–CT complexes emulsions exhibited significantly higher (*p* < 0.05) absolute ζ-potential, suggesting greater negative charge density at the interface. The increased absolute ζ-potential of emulsions led to a high energy obstacle between emulsion droplets, thereby providing good electrostatic stabilization. The increase in absolute ζ-potential is because catechins let the secondary structure of RBP change, and then protein structure becomes extended, decreasing the exposure of the positively charged groups, hence increasing the apparent negative charge of the protein (36). The pI values of EWP–CT and EWP–CA complexes were mildly lower than those of EWP, suggesting that CT and CA complexes changed the surface charge characteristics of EWP. It can be explained that CT or CA complexes possibly decreased the number of exposed positive groups on the surfaces of the protein, or increased the number of exposed negative groups (40). This is consistent with the findings of Li et al. (36).

The addition of polyphenols can induce protein aggregation and depolymerization, so the particle size of PPCs may change with the addition of polyphenols. Research showed that the average diameter of β-CG–EGCG co-assembled nanoparticles increased in the presence of EGCG. Moreover, scanning electron microscopy confirmed that the particle size gradually increased with the increase of EGCG concentration (55). Staszawski et al. (6) measured the distribution size of β-LG and tea polyphenol particles in emulsions prepared with similar materials. They concluded that β-LG formed nanocomplexes with polyphenols, with β-LG–polyphenols particles being larger than pure β-LG ones. The increase in particle size was attributed to the binding of polyphenols with proteins occurring on the hydrophobic side chain and near-plane side chain, which is mainly due to the aforementioned accumulation of polyphenol rings on the hydrophobic side chain (6). Finally, the complexes of polyphenols with proteins are evenly dispersed an oil-in-water emulsion, and have higher stability.

By measuring the droplet size of PPCs, one may obtain information on the stability of the oil-in-water emulsions containing protein–polyphenol. The particle size may be related to the pH value. Feng et al. (16) studied the particle size of oil droplets of OVA and OVA–polyphenol when the pH were 3.5 and 7. The particle size of OVA-stabilized oil droplets under the two pH values was double than that of oil droplet, which might be due to droplet aggregation and/or coalescence. However, the stable droplet size of OVA–polyphenol complexes were all smaller than that of OVA, and there was little difference between pH 3.5 and 7. This showed that the conjugation of OVA and polyphenol had beneficial stability effects against droplet aggregation and coalescence (16).

### Rheology and Gel Texture

Rheological properties are among the most important attributes of food emulsions, and are closely related to their texture, sensory properties, and shelf life (16, 32). The gelling properties of the proteins contribute substantially to the stability of the emulsion rheology. The researchers thought that the improved emulsion stability may be due to the formation of gel-like microstructure, which has been significant in stabilizing emulsions prepared by SPI and whey protein (26, 56). Emulsions stabilized by OVA showed a higher viscosity with a more distinct pseudoplastic behavior as compared with those stabilized by OVA–CT complexes, as evidenced by their higher consistency coefficient (k) but lower rheological index (n). This showed that OVA-coated emulsions were more prone to flocculate, which manifested in their larger particle size (16).

Studies have shown that water or oil crystallization will cause phase separation during freeze-thaw processes, and the increase of emulsion viscosity can prevent this phenomenon (45). Protein gels can also enhance the stability of the emulsion by forming a three-dimensional network structure (14). Many plant polyphenols are able to interact with proteins, thus improve the gelling properties (57). Gels formed by covalent interactions are more rigid and heat stable than those formed by non-covalent interactions. The complexes formed by the binding of β-LG and tea polyphenols significantly enhanced the gelation ability of β-LG, because tea polyphenols enhanced the gelation speed of the protein (28). Tea polyphenols can also increase the whey protein gel hardness or viscosity, depending on the case (58). Yan et al. (59) reported that the conjugate of GA or rutin into gelatin from walleye pollock (*Theragra chalcogramma*) skin at a concentration of 20 mg/g dry gelatin significantly increased gel strength. In addition, the viscous modulus and elastic modulus of gelatin gel were improved by the crosslinking of carboxyl group skeletal and C–N–C group of gelatin molecules with rutin or GA.

### Microstructure

Microstructure refers to the structure of substances, organisms, cells under the microscope, and also refers to structures in the nano scale, the molecular scale, the atomic scale, and even the subatomic scale. Through the electron microscope, it can be found that protein sample which is not conjugated to polyphenols exhibited a morphology characteristic without fibrillar or sheet-like structures (50). For example, soybean protein (SP) has a variety of structures which can interact with tea polyphenols to form complexes, and the microstructure of soybean protein can be observed by scanning electron microscopy (60). This work showed that the study of SP microstructure can be used to study the function of complexes in emulsions. In addition, Cryo-SEM images of soy protein isolate-anthocyanin (SPI–ACN) composite nanoparticles and emulsion showed that the SPI–ACN composite nanoparticles were spherical and had diameters ranging from 200 to 500 nm. In addition, increasing the amount of ACN at low temperatures led to the formation of highly porous structures (61). The researchers suggested that this might be due to the covalent interactions between ACN and SPI, which then untied the protein-peptide chain, then the latter interacting with adjacent droplets, eventually forming emulsions in a bridging flocculation-type induced structure (62). The structure of the bridged emulsion was the typical structure of the protein-stabilized emulsion, which can accelerate the formation of the network structure of the emulsion and thus enhanced the stability of the emulsion. In addition, the emulsion stabilized by SPI–ACN showed that the droplets dispersed were good without obvious coalescence. With the increase of ACN, the particle size of droplets decreased significantly. Moreover, it was observed that SPI–ACN adhered to the surface of emulsion droplets densely.

Furthermore, Gu et al. (3) prepared β-CG–EGCG assembled particles in an ethanol-mediated process and used this assemble complexes to stabilize a high internal phase emulsion (HIPE). Using scanning electron microscopy, they found that the spherical composite nanoparticles gradually increased when the concentration of EGCG–β-CG particles increased. From the optical microscope observation, it could be inferred that most of the droplets in these HIPEs were closely packed, and in many cases, two adjacent droplets shared the same interface layer, again in a bridging flocculation-type of structure. High internal emulsion thus showed a more homogeneous and stable state (3).

### Creaming and Flocculation

Food emulsions usually consist of aqueous (continuous) and oil (dispersed) phases (63). The presence of two phases in the emulsion causes flocculation and aggregation of the emulsion during processing, storage and transportation (64). Such temporal instability can be followed by permanent destabilization via coalescence and/or Ostwald ripening. As a result, the emulsions tend to revert to the original two phases state. The protein–polyphenol complexes are added to the emulsion as an emulsifier and adsorbed on the surface of the emulsion droplets to form a dense film. It can reduce the interfacial tension of the protein on the surface of the droplet, which reduces the coalescence rate, as well as reduces the extent of flocculation of the emulsion, thus enhancing its stability. Thus, the emulsion can be prevented from instability caused by such causes as coalescence or Ostwald ripening (15, 63). Therefore, under such interactions, the emulsification of protein and the stability of the emulsion stabilized by protein–polyphenol complexes would be improved (42, 43).

One of the key factors to produce emulsion is the density difference between lipid phase and water phase. The creaming index (CI) is known as a useful indicator in terms of quantifying the extent of separation of lipids from the water phase in an emulsion (65). It is typically a function of the density difference, particle size, continuous phase viscosity and extent of flocculation, among others. Ju et al. (65) proposed that the emulsion formed by SPI–ACN complexes nanoparticles had higher stability at room temperature, which might be due to its low CI value. Therefore, it can be considered that the enhancement of emulsion stability might be attributed to the change of protein microstructure after interacting with polyphenol, which was of significance in stabilizing SPI emulsions or whey protein and chitin emulsions (26, 56). Zhu et al. (39) studied the interaction between gliadin and TA nanoparticles (GTNP) in order to stabilize the emulsion, and measured the relevant CI. The results showed that the CI of majority of the studied emulsions increased significantly within short term and subsequently stabilized. For example, the CI of GTNP complexes appeared to plateau at 10 h, whereas the CI of the other emulsions appeared to plateau within 5 h, which indicated the slowest creaming rate of the GTNP complexes-stabilized emulsions. This is due to the observation that the particle size of gliadin–TA complexes decreased, due to the best emulsification properties of the complexes (39).

According to related studies, β-LG and CT covalent complexes formed by the free radical method can stabilize the oil-in-water emulsion more effectively than β-LG itself (18). Chen et al. (66) showed that the porcine plasma protein-CA complexes could be rapidly adsorbed to the oil surface to form an interfacial film, which indicated an improved emulsifying property of the complexes. The improvement of the emulsifying properties of the protein–polyphenol complexes can also reduce the flocculation and eventual instability of emulsions (14). Therefore, the stability of the emulsions can be conditionally enhanced by the interaction of proteins and polyphenols, thus alleviating the flocculation and coalescence of the produced emulsions.

### Antioxidant Ability

Oil-in-water emulsions are easy to be oxidized during storage and processing. This is mainly because the interaction between lipid oxidation products and amino groups on proteins can accelerate protein oxidation (67). Some researchers have reported that transition metals are the main oxidants of oil-in-water emulsions (68). In emulsions, transition metals mainly promote oxidation by decomposing lipid hydroperoxides on the droplet surface into free radicals. Some proteins are good emulsifiers, but they do not often display strong antioxidant capacity. However, after combination with polyphenols, their antioxidant capacity can be greatly enhanced (14, 50).

More and more researchers have been carrying out in-depth studies on the antioxidant properties of protein–polyphenol complexes. The ability of protein–polyphenol complexes to control droplet oxidation have been demonstrated in a number of works. The concentration of hydrogen peroxide in plain β-LG stabilized fish oil emulsion from 20 to 214meq/kg oil during the 30 days storage process (18). On the other hand, the hydrogen peroxide content of the emulsions formed by β-LG-polyphenol complexes never exceeded 40 meq/kg oil during the 30 days storage process, which suggested that β-LG and polyphenols complexes are highly efficient antioxidants. That was because β-LG and polyphenols complexes formed a surface film on the droplet interface, the adsorbed entities blocking the free radicals before entering the oil droplet surface, thus avoiding oxidation. The interface region formed by the complexes was the contact region between lipid and water, which was also the key region for the development of oxidation (69). PPCs were captured and located at this interface as antioxidants to prevent lipid oxidation.

In addition, continuous phase proteins in oil-in-water emulsion can also inhibit lipid oxidation by scavenging free radicals. Although many amino acids can participate in free radical scavenging, cysteine and tyrosine are reported to be the two most important free radical scavengers. However, proline and cysteine are the main binding sites of protein to polyphenols. Free radicals are another main factor leading to oxidation of droplets of oil-in-water emulsion. Waraho et al. (70) concluded that protein–polyphenol complexes can make an important contribution to free radical scavenging. They showed that OVA–CT complexes can scavenge free radicals and/or inactivate promoters such as transition metals at the lipid–water interface (70), thereby preventing lipid hydroperoxide (LOOH) from decomposing into alkoxyl (LO′) and peroxyl (LOO″) free radicals. In addition, the activity of these highly active free radicals, which extracted hydrogen from unsaturated fatty acid (LH) to form new free radicals was inhibited (5). In addition, compared with the OVA stabilized fish oil emulsion, the interfacial protein content (F_ip_) of the OVA–CT complexes-stabilized fish oil emulsion increased significantly, while the continuous phase protein content (F_cp_) decreased significantly. This might be due to the decrease in the interfacial tension of the protein after the CT grafting reaction, which can be due to the partial negation of the protein hydrophobicity: the covalent interactions involved the non-polar parts of the protein, leading to a stronger binding to the lipid-water interface once adsorbed (45). Therefore, the lipid droplets in the emulsion stabilized by the conjugate could have a denser and defect-free interface film, thus effectively preventing the penetration and diffusion of the oxidation initiators and thus arresting them from reacting with ω-3 polyunsaturated fatty acids.

### Thermal Stability and Salt Stability

In some situations, the thermally-induced denaturation of proteins is detrimental to the stability of emulsions (71). After heat treatment, flocculation and coalescence often occur in oil-in-water emulsion droplets which were stabilized by proteins, and thereby affected the function of bioactive substances in the emulsion. Droplet flocculation could be attributed to the unfolding of the globular proteins adsorbed onto the lipid droplet surfaces when heated above their thermal denaturation temperature (72). This will result in increased exposure of surface-active groups (such as hydrophobic and sulfhydryl groups), which will increase the attractive interaction between droplets. The PPCs can effectively protect the emulsions from heat treatment because of the interfacial film formed on the surface of the emulsion droplets. The stability of β-carotene-containing emulsion stabilized by LF and LF–polyphenol complexes was determined at 100°C for 20 min (32). The study showed that the emulsion stabilized by protein showed poor thermal stability, which could be attributed to the unfolding of the LF macromolecules. The thermal denaturation of the adsorbed globular protein led to the increase of the hydrophobicity of the lipid droplet surface, which generated a strong hydrophobic attraction between the lipid droplets. Schmelz et al. (72) showed that LF-coated emulsions formed gels when temperatures exceeded 70°C, which was because the enhancement of hydrophobic attractions induced extensive flocculation of denatured protein-coated droplets. However, the emulsions coated by LF–CA and LF–EGCG complexes were highly stable to heat, and their particle size distribution did not change much with heating. In addition, DSC results showed that protein–polyphenol complexes had higher denaturation temperatures than protein alone. Thus, the emulsion stabilized by protein–polyphenol complexes had better thermal stability. It is worth noting that, precisely because of the higher thermal stability of the covalent complexes, the covalently-modified protein was relatively easy to expand, requiring very little energy toward that aim (45). It also presented a new challenge for the study of thermal stability of modified proteins in emulsions.

Under low salt concentrations, the average particle sizes of the emulsions were relatively small, but under higher salt concentrations, the average particle sizes increase, which is related to the electrostatic shielding effect. That is, when salt is added into the aqueous phase, counterions are gathered around the charged surface groups, and the size and range of electrostatic repulsion between oil droplets are reduced (63). In addition, the effects of EWP, EWP + CT physical mixtures (not essentially interacting) and EWP–CT covalent complexes on emulsion were investigated (as mentioned above). For the emulsion stabilized by EWP alone or EWP+CT mixtures, the average particle size of the emulsion increased significantly when the concentration of NaCl increased from 50 mm to 100 mM, while for the emulsion with stable EWP–CT conjugates, the average particle size of the emulsion increased significantly only when the concentration of NaCl increased from 100 to 200 mM NaCl. These results showed that the emulsions stabilized by EWP–CT complexes had better salt stability than the other two systems, which might be due to the stronger spatial repulsions caused by the presence of polyphenols. Therefore, the oil-in-water emulsions stabilized by EWP–CT had better thermal stability and salt stability.

### Digestive Characteristics

Studies of protein–polyphenol compound interactions aim to better analyze and understand phenomena that take place during digestion (43). The oil droplets, coated by protein–polyphenol complexes, enter the gastrointestinal tract, where they are exposed to continuous alterations in shear fields, pH, enzymic, salt and surfactant environments. Digestion simulations have been extensively used in order to probe such processes (Figure 4). These studies aim to comprehend the basis of bioavailability and fat absorption process from emulsions, and to lay a foundation for the research of functional foods (73).

Jiang et al. (62) found that all protein components in casein (CS) or WPI were fragmented into small peptides during a simulated gastrointestinal pepsin–pancreatin digestion. In both CS and WPI samples, the treatment with increasing concentrations of catechin acid (CA) resulted in lighter peptide bands, suggesting improved digestibility. In addition, for all CS samples, only a few small peptides remained present in the electrophoretic graph after 1 h of simulated gastric hydrolysis and almost no peptides were found after subsequent pancreatic hydrolysis. With the increase of CA content, more and more proteins were digested into smaller peptides which were lysed after 1 h of digestion (62). It can be illustrated that the addition of CA promoted digestibility of both WPI and CS. When the emulsion drops were digested in the stomach, the pH value, ionic strength and enzyme activity of the environment of the gastrointestinal would change, and the particle sizes of the emulsions showed an increasing trend in the gastric digestion stage (7). In the stomach stage, due to the relatively low gastric pH value, the absolute value of the ζ-potential of the emulsion decreased, thus reducing the electrostatic repulsion between the lipid droplets, promoting flocculation. Microscopy images showed that the droplets formed by mixtures of LF and LF–EGCG was more physically stable under this environment, and the droplet formed by the covalent complexes was less stable and the droplets were of uniform size distributions. The results showed that the covalent bonds of EGCG and LF could improve the stability of the systems during the digestive process. The absolute value of the ζ-potential of the three emulsions was increased, so the electrostatic repulsion between emulsion droplets also increased. Microscopic image suggested that LF–EGCG covalent complexes can render oil-in-water emulsions more stable during intestinal digestion.

In addition, the extent of emulsion destruction depends on the amount of free fatty acids released during intestinal digestion (65). The amount of free fatty acids released from protein–polyphenol emulsions was generally used to reflect the decomposition of oil-in-water emulsion during digestion. Studies have shown that LF–EGCG covalent complexes can delay the release of fatty acids to some extent when *in vitro* measuring the fat digestibility and digestibility of different samples in small intestine by automatic pH titration (7). In the same way, Zhou et al. (74) also reported that the free fatty acid released from emulsions stabilized by gliadin were restrained when proanthocyanins were added, which is because proanthocyanin also found their way at the interface along the proteins. Moreover, studies showed that the release rate of free fatty acids in fish oil emulsions stabilized by gliadin-grape seed proanthocyanidins (GSP) complexes was lower than that of normal emulsions. It can be concluded that the digestion time of normal emulsion was 20 min, and that of complexes stabilized emulsion was longer, with free fatty acid release rates of 92.5 and 63.3%, respectively (49). The results showed that the digestibility of emulsion stabilized with gliadin–GSP was lower than that of free gliadin.

### Nutrient Delivery Capacity

**Figure 3:**
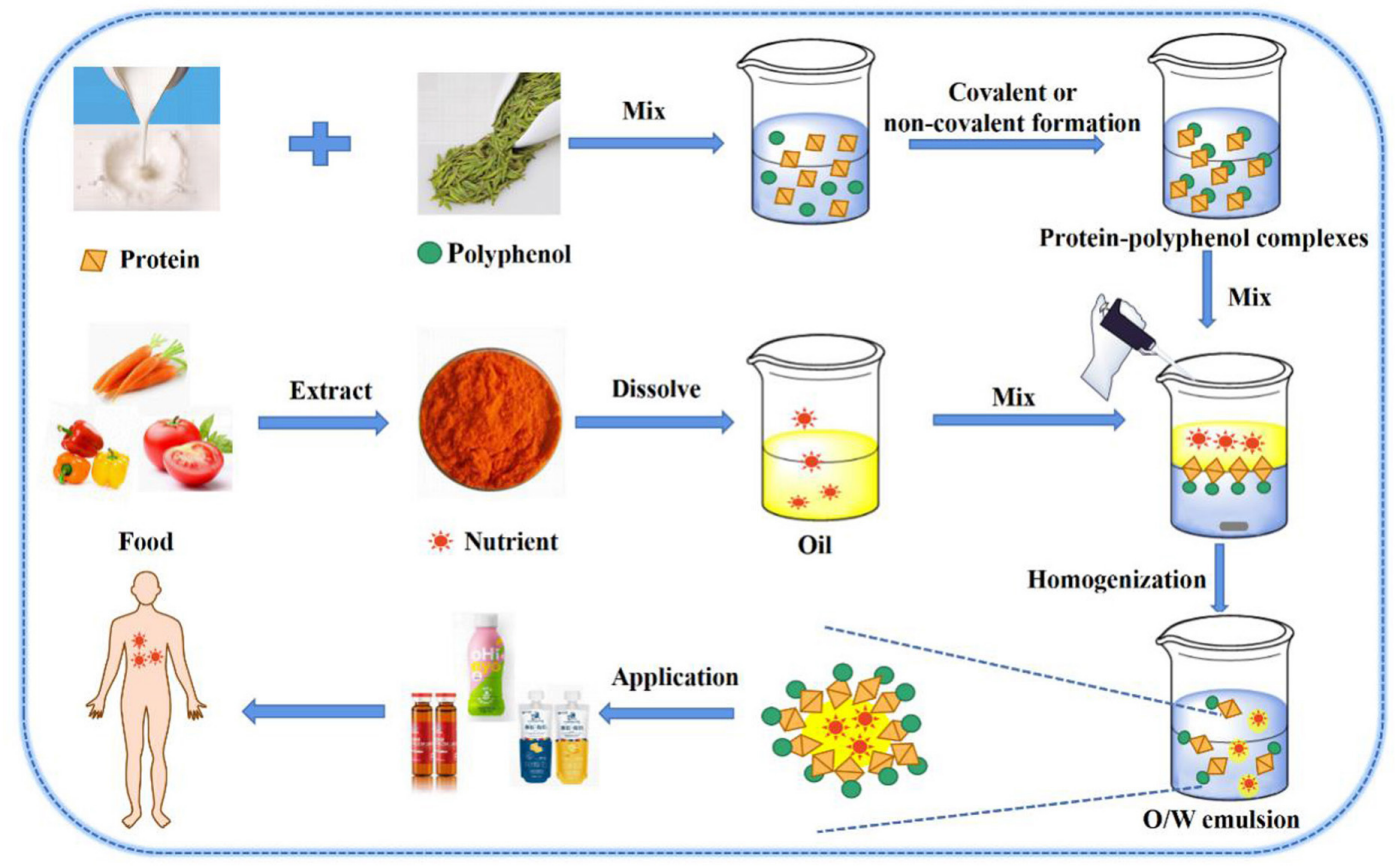
O/W emulsion nutrient delivery system: Protein-polyphenol complexes load nutrients in O/W emulsion.

Oil-in-water emulsion delivery systems are potentially competent of delivering functional oils or lipophilic active substances, which can improve their solubilities and bio-availabilities, and availably control release (Figure 3) (75). PPCs can be adhered to oil-in-water (O/W) interfaces, supplying emulsions with both protein surface activity and polyphenol antioxidant activity. Recently, protein-polyphenol complexes have shown that they possessed the binding affinity toward both hydrophobic and hydrophilic compounds because of the amphiphilic properties of their protein parts (76). Previous studies have shown that PPCs can be utilized as carriers for liposomes (77), micelles (78), and cyclodextrin/lipid complexes (79). These have been aroused interest in the application of protein–polyphenol complexes-based emulsifiers to create emulsion-based delivery systems for encapsulation of bioactive compounds such as carotenoids, curcumin, and lutein (15). In addition, this emulsion can be used in yogurt, beverage, and iron supplements and other products, and has good prospects for development.

**Figure 4:**
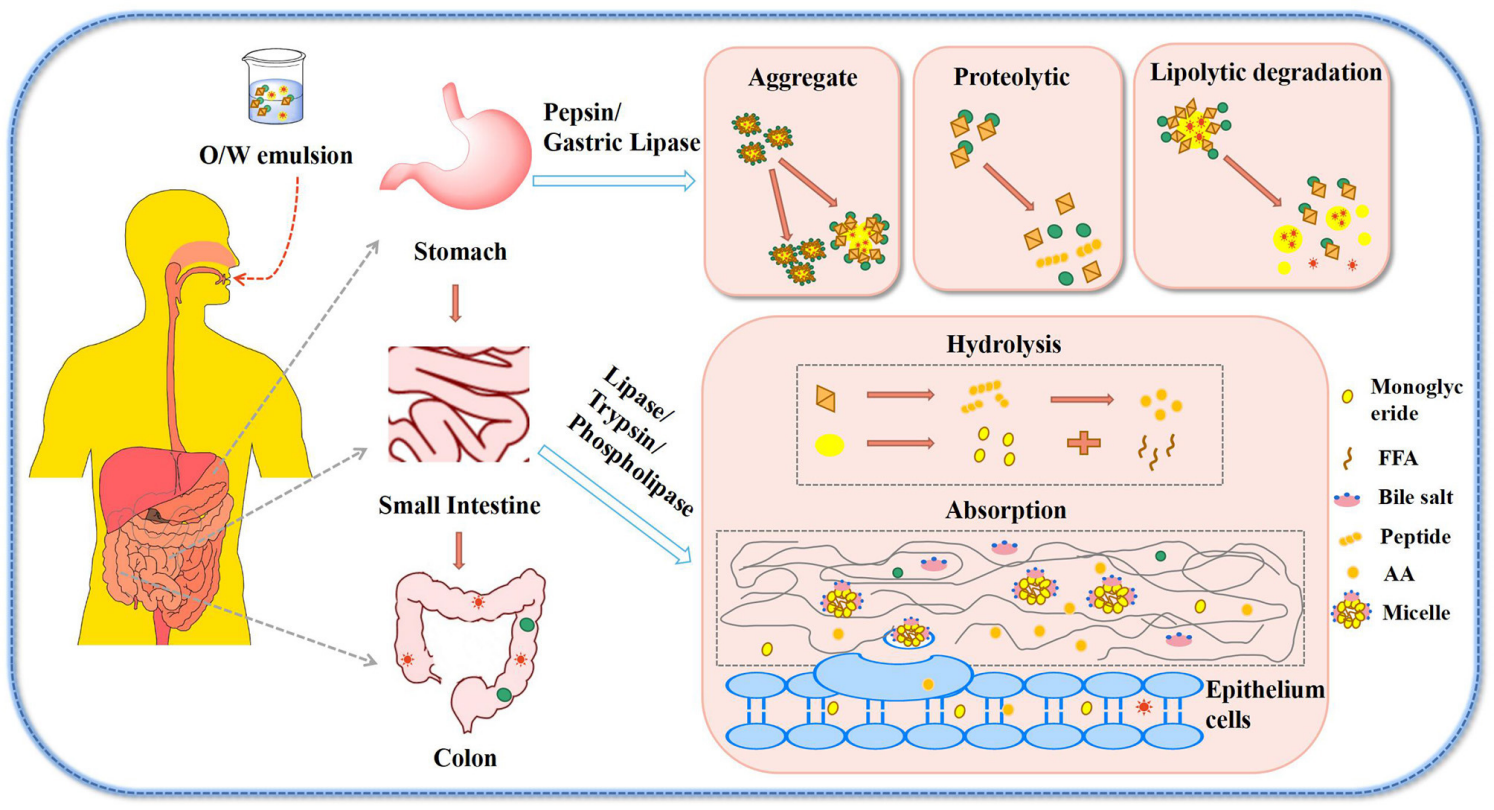
Digestive behavior of protein-polyphenol stabilized O/W emulsion.

It is well-known that β-carotene has been widely studied in industry and academia because of its unique antioxidant and vitamin A-promoting bioactivity. People have tried to design delivery systems of β-carotene as to improve its dispersion and chemical stability, and finally enhance its functionality (Figure 4). At the same time, oil-in-water emulsions have been proved to be effective delivery systems for fat-soluble bioactive components. β-Carotene stabilization was been reported in oil-in-water nano-emulsions by conjugate-based delivery systems such as α-lactalbumin–CT (18), β-LG–CT (19), and LF–CT (32). Gu et al. (42) studied and determined the ability of EWP–CT complexes synthesized by a free radical method to form and stabilize β-carotene emulsion transport systems. The results showed that the particle sizes of β-carotene emulsion stabilized by EWP–CT increased more than those of EWP. It can be assumed that the EWP–CT complexes produced smaller droplets during the homogenization process, mainly because the complexes reduced the interfacial tension of the protein. Such processes are also known to favor droplet re-coalescence and destruction during homogenization (80). Therefore, the fact that the use of EWP–CT complexes led to larger droplets was probably due to other factors. First of all, the adsorption rate of EWP–CT complexes products to the droplet surface during homogenization could be slow because of their high molecular weight or their surface chemistry change, which might lead to more re-coalescence in the homogenizer. Secondly, the properties of the interface layer formed by EWP–CT complexes, such as their thickness, hydrophobicity or charge, might change the aggregation stability of droplets. For example, the formation of complexes might change the colloidal interactions between droplets. The complexes of polyphenols with proteins could also increase the hydrophobic attraction between droplets. On the contrary, the covalent binding of polyphenols with proteins should increase the thickness of the interface coating, thus increasing the spatial repulsion between droplets. The differences in particle size and potential of different proteins and polyphenols have been described above. The average particle size and stability of each emulsion was related to the type of protein and polyphenol.

Moreover, Yi et al. (19) also improved the retention of β-carotene in emulsions by free radical methods. The Z-average diameters of β-carotene emulsion coated with α- LA (158.8 nm) and α-LA–CT (162.7 nm) were different. The PDI values of α-LA and α-LA-CT stabilized nano-emulsions were 0.109 and 0.118, respectively. Compared with the α-LA ζ-potential of −57.5 (*P* < 0.05), the ζ-potential of α-LA–CT complexes was as low as −61.9 (*P* < 0.05), which was consistent with the results of Gu et al. (42). This was due to the negative charge of catechins at neutral pH. Because the density of β-carotene-loaded oil droplets was quite different from that of dispersions, the nanoparticles that tended to rise and aggregate on the surface may be excited by gravity, which might be due to the great difference in density between β-carotene-loaded oil droplets and dispersions, so it could be caused by gravity. Nano-emulsions are essentially thermodynamically stable colloidal dispersions. The diameter stability of nano-emulsion is very important for its application in food, because its texture, appearance and taste depend on the stability of structure. In addition, temperature might be an important factor determining the physical stability of β-carotene nano-emulsions (18). It has also been reported that the diameter increment of nanoparticles or emulsions increases with increasing temperature, which was due to the fact that the temperature effect leads to faster movement at higher temperatures, thus increasing gravity flocculation (19, 81).

It was also found that α-LA–CT binding strongly inhibited the degradation of β-carotene in the emulsion at different temperatures. For example, the nano-emulsion prepared with α-LA–CT conjugates had only 4.4% β-carotene degradation in the first 2 days at 50°C, and 71.3% β-carotene degraded after 16 days, while at the end of the measurement (30 days at 50°C), there was still 56.7% β-residual carotene in the sample. α-LA–CT complexes had excellent antioxidant activity, and its mechanism was proposed: The complexes had a good ability to scavenge hydroxyl radicals and protect β-carotene from degradation. Secondly, the natural transition metal (Fe^3+^) in aqueous phase might be the main factor leading to the oxidation and degradation of β-carotene (81). The protein–polyphenol complexes can be used as powerful metal ion binders and reducers, as shown by the relevant results, which could inhibit Fe^3+^ from its role as an oxidizer, and protect β-carotene from degradation. In short, compared with the nutrient emulsion based on protein, the emulsion loaded with protein-polyphenol complexes had a strong nutrient transport capacity, less loss and strong stability.

## Conclusions

In this paper, the interaction mechanism between protein and polyphenols was reviewed, initially being divided into covalent interaction and non-covalent interactions. Hydrogen bond and hydrophobic interactions are the main driving forces for the formation of such protein-polyphenol complexes (PPCs). The interactions between proteins and polyphenols affect the structural and functional properties of proteins. The PPCs can reduce the number of proteins α-helix and change the interfacial properties of protein. The interface region formed by the PPCs on the surface of oil droplets is essentially the contact region between lipid and water. The change of the interfacial properties of the emulsion affects the emulsification, thermal stability and oxidation resistance of the emulsion, thus affecting the function of the complexes in the emulsion. Studies have shown that when protein and polyphenol are complexed, a layer of film forms on the surface of oil droplets, thus preventing the entry of free radicals and some metal ions, and improving the stability of emulsion oxidation.

Moreover, the size of oil droplets coated with PPCs is small, which suggests prevention of coalescence and flocculation of the oil droplets, thus improving the emulsion stability. In addition, the PPCs could make the particle size of the emulsion smaller, which improves the bioavailability of nutrient. Furthermore, PPCs play an important role in the process of lipid to nutrient delivery. As a broad rule, the lipid delivery capacity of oil-in-water emulsions is enhanced in the presence of PPCs, while the retention rate of nutrients is higher. Through this study, it can be concluded that protein–polyphenol complexes play an important role in improving the stability, digestion characteristics and the ability of nutrient delivery of oil-in-water emulsions. This paper can provide guidelines for enhancing the application potential of PPCs, especially in improving stability and nutrients delivery of oil-in-water emulsion system in the food industry.

## Author Contributions

ML: conceptualization and writing original draft. CR, JL, QD, and WL: writing—review & editing and supervision. YL and YD: supervision. All authors contributed to the article and approved the submitted version.

## Funding

This work was financially supported by the National Natural Science Foundation of China (Grant No. 31972104) and National Key R&D Program of China (2019YFD0901603).

## Conflict of Interest

YL was employed by company Hangzhou Huadong Medicine Group Pharmaceutical Research Institute Co. Ltd. The remaining authors declare that the research was conducted in the absence of any commercial or financial relationships that could be construed as a potential conflict of interest.

## Publisher's Note

All claims expressed in this article are solely those of the authors and do not necessarily represent those of their affiliated organizations, or those of the publisher, the editors and the reviewers. Any product that may be evaluated in this article, or claim that may be made by its manufacturer, is not guaranteed or endorsed by the publisher.

## Abbreviations

ABTS, 2: 2'-azino-bis (3-ethylbenzothiazoline-6-sulfonic acid)
ACN: Anthocyanin
LO^·^: Alkoxyl
β-LG: β-lactoglobulin
BSA: Bovine serum albumin
C: (+)-catechin
CA: Catechin acid
CD: Circular dichroism
CI: Creaming index
CS: Casein
CT: Catechin
DHA: Docosahexaenoic acid
DPPH, 1: 1-diphenyl-2-picrylhydrazyl
DSC: Differential scanning calorimetry
EAI: Emulsifying activity index
EGC: (-)-epigallocatechin
EGCG: (-)-epigallocatechin gallate
EPA: Eicosapentaenoic acid
ESI: Emulsion stability index
FPP: Flaxseed polyphenols
FPI: Flaxseed protein isolate
FRAP: Ferric reducing activity power
GA: Gallic acid
GSP: Gliadin-grape seed proanthocyanidins
GTNP: Gliadin and TA nanoparticles
HIPE: High internal phase emulsion
LF: Lactoferrin
LOOH: Lipid hydroperoxide
MALDI–TOF–MS: Matrix-assisted laser desorption/ionization time-of-flight mass spectrometry
O/W: Oil-in-water
ORAC: Oxygen radical absorbance capacity
OVA: Ovalbumin
LOO^··^: Peroxyl
PPCs: Protein–polyphenol complexes
RBP: Rice bran protein
SDS–PAGE: Sodium dodecyl sulfate-polyacrylamide gel electrophoresis
SP: Soybean protein
SPI: Soy protein isolate
TA: Tannin acid
TBARS: Thiobarbituric acid reactive substance
Td: Decomposition temperature.
